# Experimental Study on the Response Mechanisms of Drift Egg Transport and Adhesive Egg Hatching to Reservoir Impoundment in the Lower Jinsha River

**DOI:** 10.3390/ani15172488

**Published:** 2025-08-25

**Authors:** Lekui Zhu, Wenchao Li, Dong Chen, Yiheng Gao, Rui Han

**Affiliations:** 1 Key Laboratory of Water Cycle and Related Land Surface Processes, Institute of Geographic Sciences and Natural Resources Research, Chinese Academy of Sciences, Beijing 100101, China; zhulk.16b@igsnrr.ac.cn; 2 College of Resources and Environment, University of Chinese Academy of Sciences, Beijing 100049, China; 3 College of Life Sciences, Shihezi University, Shihezi 832003, China; lwc1464347092@163.com; 4 State Key Laboratory of Water Cycle and Water Security in River Basin, Beijing 100038, China; wkbnjy@163.com (Y.G.); hanrui_first@163.com (R.H.)

**Keywords:** microtopography, sediment deposition, restart, reproduction, fish eggs

## Abstract

Understanding the impoundment effects of cascade reservoirs on fish reproduction is essential for the conservation and management of river ecosystems. Using the lower Jinsha River as an eco-hydraulic reference, this study conducted laboratory experiments to investigate how hydrodynamic–microtopography interactions influence the near-bed transport of drifting fish eggs and how sediment deposition affects the hatching success of adhesive demersal eggs. A predictive formula for near-bed egg drift was established, and a novel threshold for near-bed drift was proposed. In a separate set of experiments, sediment deposition was found to significantly reduce the hatching success of adhesive demersal eggs—specifically *Schizothorax prenanti* and *Procypris rabaudi*—primarily by decreasing dissolved oxygen levels (*p* < 0.05). These findings provide a scientific basis for improving reservoir operation strategies and mitigating the ecological impacts of sedimentation on fish reproduction in impounded rivers.

## Simple Summary

Cascade reservoirs, like those built along the lower Jinsha River, can have serious impacts on fish reproduction. To understand these effects, we carried out lab experiments based on the conditions of this river. First, we studied how water flow and small changes in riverbed shape affect the movement of drifting fish eggs near the bottom. Then, through another set of experiments, we looked at how sediment siltation can reduce the hatching success of fish eggs that stick to the riverbed. We found that too much sediment can lower oxygen levels in the bed, making it harder for certain fish species—like *Schizothorax prenanti* and *Procypris rabaudi*—to hatch successfully. Our findings can help improve how we manage reservoirs and protect fish in dammed rivers.

## 1. Introduction

The impoundment and operation of reservoirs have significantly altered the hydrodynamic environments and bed characteristics of natural rivers, particularly forming typical “backwater zones” with reduced flow velocity and increased sediment deposition in the reservoir impoundment area [1,2,3,4,5,6]. This environmental transformation has profoundly impacted fish reproductive processes. In recent years, with the successive operation of large hydropower stations, i.e., Xiluodu, Xiangjiaba, Baihetan, and Wudongde, the lower Jinsha River has gradually transformed from a typical mountainous river into a human-regulated river with controlled flow velocity and a tendency toward sedimentation [5,7,8,9,10]. In certain reaches—particularly fluctuating backwater zones or confluence areas—flow velocity is markedly reduced despite limited changes in water level. As a result, bed sediment becomes significantly finer, accompanied by microtopographic restructuring, which seriously disrupts fish spawning habitats [7,8,9,10].

According to their physical characteristics, fish eggs can be classified into two main types: drifting eggs and adhesive, depositional eggs [6]. Drifting fish eggs depend on flowing water to remain suspended during development, and their hatching success is influenced by flow velocity, hydrological connectivity, and the length of the river segment [11,12]. Reservoir impoundment disrupts the natural riverine hydrodynamics, creating stagnant or low-velocity zones in certain reaches [1,13,14] and constraining the transport pathways and oxygen exchange for drifting fish eggs, ultimately impairing their hatching success [6,11,15].

Current research on drifting fish eggs primarily focuses on their transport and deposition in the surface layer of flows, with a common assumption that eggs settling to the riverbed are non-viable or dead [11,12]. Most survival thresholds for drifting fish eggs refer to the flow conditions at which eggs begin to settle out of suspension, determined by the balance between gravitational settling forces and upward turbulent forces [16,17,18,19,20]. However, this assumption may oversimplify the fate of eggs in natural environments. Recent findings suggest that the settlement of drifting fish eggs onto the bed, or even temporary burial by sediment, does not necessarily result in immediate mortality [21]. Greater attention should be given to the “near-bed drift” mechanism and newly defined survival threshold for drifting fish eggs in the subsurface and bottom water layers.

In contrast to drifting eggs, adhesive demersal fish eggs typically attach to substrate surfaces or become embedded within gravel layers during incubation, rendering them more sensitive to benthic environmental conditions [6]. In addition to hydrodynamic factors, their hatching process is influenced by various physicochemical parameters such as dissolved oxygen (DO), light intensity, and pH [22,23]. However, impounded river sections often experience accumulation or coverage of fine sediments [16,17,18,19,20,21], which can reduce oxygen levels and light penetration in the water column, thereby adversely affecting the egg incubation process [24,25,26]. As a result, different types of fish eggs exhibit divergent responses to the environmental alterations induced by reservoir impoundment.

In addition, although some studies have investigated the effects of sediment accumulation on the hatching of adhesive eggs, they often overlook the complexity of natural riverbed substrates and do not thoroughly examine the intrinsic relationships among sediment grain size, hatching success, and responses to DO [27,28]. These limitations hinder a comprehensive understanding and quantitative evaluation of the effects of reservoir impoundment on the hatching processes of demersal fish eggs.

To address the above research gaps, this study focuses on how hydrodynamic–microtopography coupling influences the near-bed transport of drifting fish eggs. Additionally, this study examines how sedimentation affects the hatching success of adhesive demersal eggs and explores its relationship with DO levels. The objective is to elucidate the ecological mechanisms governing fish egg hatching under reservoir impoundment conditions and to provide a theoretical basis and technical support for fishery resource conservation and ecological restoration in cascade reservoir systems.

## 2. Materials and Methods

Using the lower Jinsha River as an eco-hydraulic reference, this study designed two types of indoor experiments, i.e., flume experiments to simulate the transport of drifting eggs under varying impoundment conditions and hatching tank experiments to simulate the impact of sediment deposition on the hatching of adhesive eggs.

### 2.1. Experimental Setup

#### 2.1.1. Hydrodynamics–Microtopography Simulation Flume

**Figure 1:**
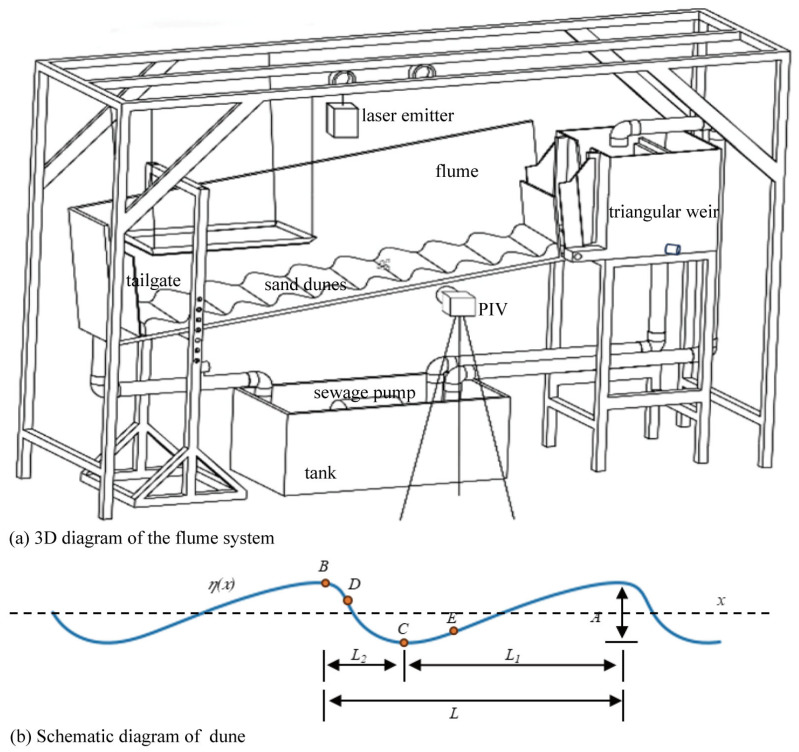
Three-dimensional view of the flume system (**a**) and schematic diagram of the dune (**b**). Note: A is the amplitude, representing the vertical distance from the dune crest to the trough (m); L is the wavelength, representing the horizontal distance between adjacent crests or troughs (m); L_1_/L_2_ is the length ratio controlling the symmetry between the stoss side and the lee side; B and C are the crest and trough, respectively, whose positions control the horizontal positions of the dunes; and D and E are the key points that control the profile shape of the dune.

To simulate impounded river reaches and investigate the mechanisms by which hydrodynamic conditions and bed microtopography affect the near-bed drifting of fish eggs, this study developed a hydrodynamics–microtopography simulation flume (Figure 1a). The system comprises a main tank (0.5 m × 0.5 m × 0.5 m, equipped with a water level control board and flow straightening grid), a triangular weir, a flume section (2 m long, 0.15 m wide, and 0.8 m high, containing a flow straightening grid, an observation section, and a tailgate). Additional components include a slope adjustment apparatus and a water circulation setup powered by a WQD15-15-1.5 sewage pump (15 m^3^/h, SHIMGE, Shanghai, China). The slope of the flume can be adjusted between 0% and 15%. The observation section (1 m in length) features ultra-clear glass sidewalls for video capture and is paved with artificial dunes on a flume bed (Figure 1b).

Flow velocity fields were recorded using Particle Image Velocimetry (PIV) (Figure 1a). The PIV system comprises a high-speed camera (IDT OS10, 200 frames/min, Integrated Device Technology, Inc., Pasadena, CA, USA) and a laser emitter (LWPIV-H, 532 nm, 100 mJ/pulse, Beijing Laserwave OptoElectronics Tech. Co., Ltd., Beijing, China). Tracer particles (hollow glass spheres with a median diameter of 50 µm and a density of 0.9–1.0 g/cm^3^) were added to the reservoir tank. PIV images were processed using the Joy Fluid Measurement software version 3.0 (https://www.pivchallenge.org/, accessed on 23 January 2025) to calculate particle velocities and obtain the time-averaged flow field distribution [29].

Artificial dunes (constructed from 25 k foam) were fixed onto the flume bed. To prevent image overexposure caused by laser reflection, the dune surfaces were coated with matte black paint and covered with sand particles (median diameter *D*_50_ = 0.9 mm). The shape of the dune model was defined by the following equation [30]:

(1) $$\eta (x)=A\sin\left\{-\frac{2\pi x}{L}+\left(1-F\right)\left[a\left(\frac{\eta }{A}\right)+b\right]\right\}$$

where *A* is the amplitude, representing the vertical distance from the dune crest to the riverbed (m); *L* is the wavelength, representing the horizontal distance between adjacent crests or troughs (m); *F* is a coefficient related to bedform regimes, with a representative value of 0.2 suggested for dunes (Guo et al., 2023 [30]); *a* is the parameter that controls the horizontal location of crests and troughs (marked as *B* and *C* in Figure 1b, respectively); *b* is the parameter that determines the profile shape of the dune (related to the positions of *D* and *E* in Figure 1b); and both *a* and *b* > 0. The parameters *A*, *L*, *a*, and *b* are governed by the prevailing flow conditions and sediment characteristics.

Two types of dunes (i.e., large and small) were designed for this study, with the large dune having twice the length and amplitude of the small dune. Their specific parameters are listed in Table 1.

#### 2.1.2. Hatching Apparatus

**Figure 2:**
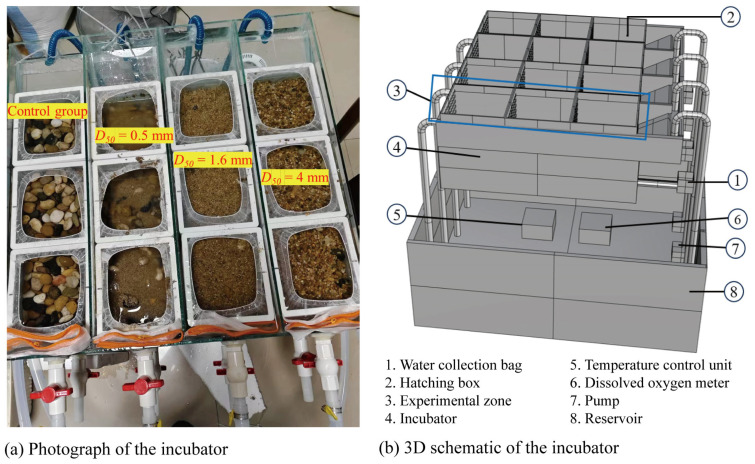
(**a**) Photograph and (**b**) 3D schematic of the incubator. Note: All pebbles/gravel and fine-grained sediments were collected in the field and sieved. We selected three distinct sediment grain sizes to represent depositional conditions at different locations within the reservoir reach.

To simulate sediment deposition environments and investigate their effects on fish egg incubation, this study designed a specialized incubation system. The system consisted of a hatching chamber, a water reservoir, a pumping device, and a seepage water collection unit (Figure 2). It provides a controlled low-flow environment, maintains a stable water temperature, and enables continuous monitoring of DO variations induced by sediment deposition.

The hatching chamber was divided into multiple independent hatching boxes with a permeable base and sidewalls. Water was introduced from one side of the chamber and discharged from the opposite side to simulate a natural, slow-flowing aquatic environment. A seepage collector was installed at the bottom of each hatching box to collect percolated water during incubation for subsequent DO analysis. DO levels were measured using a JPB-607A dissolved oxygen meter (Manufacturer: Shanghai INESA Scientific Instrument CO., Ltd., Shanghai, China., measurement error ±0.3 mg/L). A built-in temperature control unit (heater and cooler), model GH-120 Chiller—Heater (Manufacturer: SUNSUN Group CO., Ltd., Zhoushan, China), was installed in the water reservoir to maintain desired thermal conditions.

To provide a reliable baseline for assessing the effects of fine sediment deposition, a control group was established using pebble substrates with a median grain size (*d*_50_) of 25 mm (particle diameter range: 20–30 mm) without fine sediment burial (Figure 2). These substrates simulate gravel-bed conditions commonly observed in the natural spawning habitats of benthic egg-laying fish in the lower Jinsha River [31], excluding the larger cobbles and exposed bedrock occasionally encountered in the field. The absence of fine sediment in the control treatment enabled direct comparisons with sediment-covered groups, thereby isolating the specific influence of sediment deposition on incubation performance under controlled conditions.

#### 2.1.3. Experimental Materials

**Figure 3:**
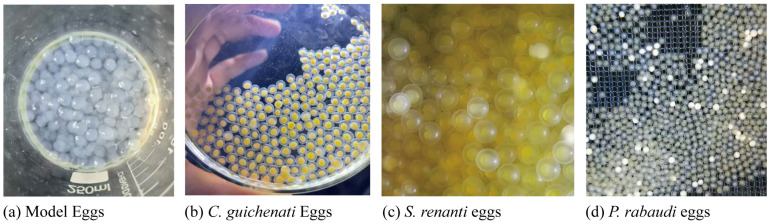
(**a**) Model eggs, (**b**) drifting eggs (*C. guichenati* eggs), (**c**) adhesive demersal eggs (*S. prenanti* eggs), and (**d**) adhesive demersal eggs (*P. rabaudi* eggs).

Considering the extended duration of the experiment and the need to eliminate potential interference from embryonic development, agar was used to fabricate model eggs for the drifting experiments. The model eggs had diameters of 5 mm and 6 mm, with density around 1.01 g/cm^3^ (Figure 3a). These physical properties closely resemble those of natural drifting fish eggs in the upper Yangtze River from Yibin to Fengdu (Figure 3b), which have similar densities and typically range from 4 to 8 mm in diameter [32]. To ensure that the model eggs exhibited settling characteristics similar to those of natural eggs, a still-water settling experiment was conducted. If the settling velocities of both are similar, it indicates that the model eggs are physically representative of real ones [33,34]. The experiment was based on the free settling velocity of spherical particles [35]:

(2) $$V=k\sqrt{\frac{\left(\rho_{g}-\rho_{w}\right)gD}{\rho_{w}}}$$

where *V* is the settling velocity (m/s); *ρ_g_* is the egg density (g/m^3^); *ρ_w_* is the water density (g/m^3^); *g* is the gravitational acceleration, taken as 9.8 m/s^2^; *D* is the egg diameter (m); and *k* is the coefficient.

The settling experiment used a transparent acrylic column with dimensions of 0.2 m × 0.1 m × 0.8 m filled with still water. A ruler was affixed to the inner wall of the column to precisely track the vertical position of the model eggs. During the experiment, the model eggs were gently placed 2 cm below the water surface and released without any initial velocity, allowing their natural settling process to be observed. A black backdrop was placed behind the column to enhance visibility. For each type of model egg, the experiments were repeated 50 times to ensure data reliability, and the results are presented in Section 3.1.

In the hatching experiments, fertilized eggs of *S. prenanti* and *P. rabaudi* were used as research subjects, provided by the Xuanhua Aquaculture Farm in Cuiping District, Yibin City (Figure 3c,d). Both species are adhesive-egg-producing fish, whose eggs exhibit varying degrees of stickiness, making them prone to settling in gravel crevices or adhering to substrates. As endemic species of the upper Yangtze River, *S. prenanti* and *P. rabaudi* were once widely distributed throughout the Jinsha River basin. However, the construction of cascade reservoirs in the reach has severely impacted their habitats, leading to a dramatic population decline [36,37]. These species have thus become representative targets for riverine ecological restoration and ecological regulation of reservoirs [38,39].

### 2.2. Experimental Methods

#### 2.2.1. Near-Bed Drift Threshold for Drifting Eggs

The experimental design involved three variables: slope (6.07%, 8.20%, 10.79%, 13.03%, and 15.45%), egg diameter (5 mm and 6 mm), and dune size (large and small), resulting in 20 distinct experimental scenarios (Table 2). Each scenario was conducted twice, producing a total of 40 sets of data. All experiments were documented using video recording.

During the experiment, once the desired hydrodynamic scenario was reached, 100 eggs were gently released onto the bed surface. In this study, the near-bed drift threshold is defined as follows: within 20 s, more than 75% of the eggs within the observation window are able to escape the circulation cells induced by sand dunes and drift downstream. The corresponding flow condition is defined as the near-bed drift threshold. Below this flow threshold, drifting fish eggs have no chance to survive. The 20 s observation period was chosen based on the possibility that, in the real world, bedforms (dunes) may shift and bury the eggs within this time frame. Previous studies have shown that once fish eggs are buried, their hatching rate decreases significantly [21,27]. Clearly, the near-bed drift threshold is lower than the traditional flow threshold required to keep drifting eggs from settling.

Experimental data were analyzed using dimensional analysis based on the Π theorem to develop a mathematical model for predicting the near-bed drift threshold of drifting eggs. Quantile Regression was applied to fit the relationship between the near-bed drift ratio and the velocity threshold at different quantiles (e.g., 5%, 25%, 50%, 75%, and 85%). To evaluate the overall fitting performance, least squares regression was also conducted.

#### 2.2.2. Effect of Sediment Deposition on the Hatching of Adhesive Eggs

Environmental parameters were used to strictly control the experiments to ensure consistency across treatment groups in terms of water depth, temperature, pH value, and other conditions. For *S. renanti*, the water temperature was maintained at 15–18 °C, and for *P. rabaudi*, at 20–22 °C. The pH ranged from 7 to 7.5, DO at the inlet exceeded 7 mg/L, and the water depth was kept at 5 cm. A water pump was used to maintain a stable, clean, low-flow environment (velocity < 5 cm/s).

Each experimental treatment was conducted in triplicate to ensure data reliability and reproducibility. A 10 cm thick layer of gravel (*d*_50_ = 25 mm) was laid at the bottom of each incubation box to simulate natural spawning substrates [40]. Each incubation box contained 100 fertilized eggs, which naturally settled and fell into the pore spaces of the gravel under the influence of water flow. Subsequently, sediments of different particle sizes were added to the gravel surface of the experimental groups (except for the control group) to simulate the sedimentation observed at various locations within the backwater reach.

The incubation duration for both *S. renanti* and *P. rabaudi* was 7 days, at which point both species’ eggs had fully hatched. Throughout the experiment, DO levels in the sediment deposition area were measured twice a day, in the morning and evening, using a JPB-607A dissolved oxygen meter (measurement error ±0.3 mg/L).

At the end of the experiment, the number of eggs that hatched normally and those that produced deformed larvae in each experimental group were recorded. These data were used to analyze the impact of sediment deposition on hatching success and embryo development quality through a mediation effect analysis.

Mediation effect analysis is used to explore whether the effect of an independent variable (X, e.g., sediment deposition) on a dependent variable (Y, e.g., hatching success or embryo quality) is transmitted through a mediator variable (M, e.g., dissolved oxygen or other intermediate factors). This helps to clarify the underlying causal pathways.

The classical approach involves estimating three regression equations. The significance threshold for each regression model was set at *p* < 0.05, and effects meeting this criterion were considered statistically significant.

Total effect of *X* on *Y*:

*Y* = *cX* + *e*_1_*Y* = *cX* + *e*_1_*Y* = *cX* + *e*_1_ (3)

Effect of *X* on mediator *M*:

*M* = *aX* + *e*_2_*M* = *aX* + *e*_2_*M* = *aX* + *e*_2_ (4)

Effect of *X* and mediator *M* on *Y*:

*Y* = *c′X* + *bM* + *e*_3_*Y* = *c′X* + *bM* + *e*_3_*Y* = *c′X* + *bM* + *e*_3_ (5)

where *c* is the total effect of *X* on *Y*; a is the effect of *X* on *M*; *b* is the effect of *M* on *Y* controlling for *X*; *c′* is the direct effect of *X* on *Y* controlling for *M*; and *e*_1_, *e*_2_, *e*_3_ are error terms.

The indirect (mediated) effect is quantified as

*ab* = *a* × *b* (6)

The total effect *c* equals the sum of the direct effect and indirect effect:

*c* = *c′* + *ab* (7)

Statistical significance of the mediation effect *ab* can be tested using methods such as the Sobel test or bootstrapping.

## 3. Results

### 3.1. Settling Velocity of Model Eggs

**Figure 4:**
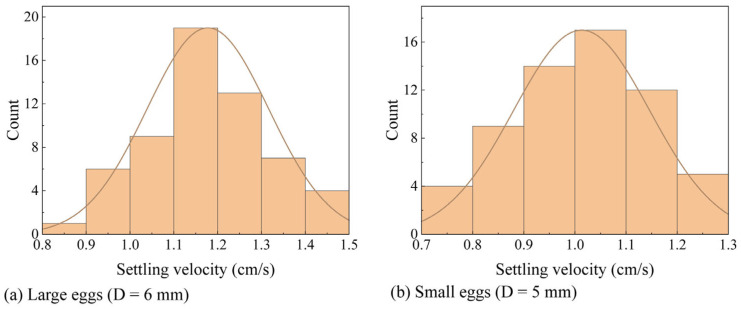
Probability distributions of settling velocity of (**a**) large eggs (D = 6 mm) and (**b**) small eggs (D = 5 mm).

Figure 4 illustrates the frequency distributions of the settling velocities of model eggs. The distributions were plotted as histograms and fitted with normal distribution curves.

As shown in Figure 4, 53% of the model eggs with a diameter of 6 mm settle at velocities between 1.2 and 1.3 cm/s, with an average of 1.178 cm/s. The model eggs with a diameter of 5 mm had an average settling velocity of 1.013 cm/s, with 50% of the model eggs settling at velocities between 0.9 and 1.1 cm/s.

Table 3 compares the *k* values obtained from our experiments, i.e., 0.553, with those from previous studies. Our experimental settling velocities are close to the field-measured values in the upper Yangtze River from Yibin to Fengdu [40]. The slightly lower values observed in our study may be attributed to the more spherical shape of the experimental eggs.

### 3.2. Near-Bed Drift Threshold of Drifting Eggs

The key parameters influencing the near-bed drift threshold (denoted as *ξ*) of drifting eggs likely include flow velocity, physical properties of the eggs (such as diameter and specific gravity), properties of the water body (such as density), and the turbulence and fluctuation characteristics of the flow:

(8) $$\xi =\int \left(U_{b},D,R_{e},r_{s},r,g,\lambda ,hsr_{s}\right)$$

According to the Π theorem [42], the following dimensionless expression can be obtained:

(9) $$\xi =\alpha \frac{{U_{b}}^{2}DR_{e}}{\frac{r_{s}-r}{r}g\lambda h_{s}}$$

where ξ is the near-bed drift threshold; *α* is an empirical coefficient; *U_b_* is the near-bed flow velocity; *D* is the egg diameter; *R_e_* is the Reynolds number; *r_s_* and *r* are the specific gravity of the eggs and water, respectively; *g* is the gravitational acceleration; and *h_s_* and *λ* are the height and wavelength of the sand dunes.

**Figure 5:**
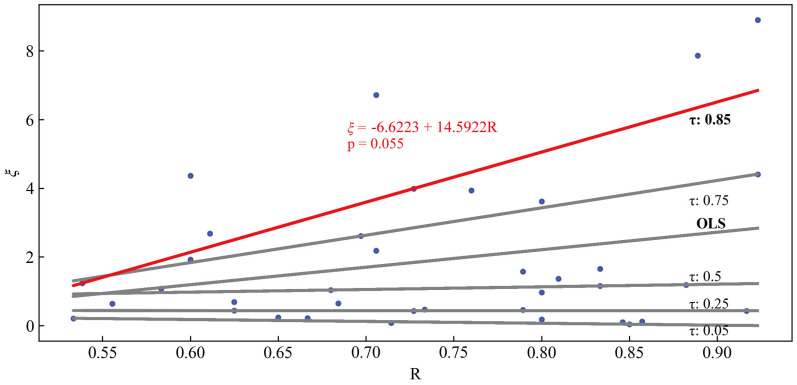
Relationship between egg reactivation proportion and threshold.

In this study, the Quantile Regression method was used to fit the relationship between *ξ* and the drift ratio *R* (the ratio of the number of near-bed drifting eggs that can drift downstream within 20 s to the total number of eggs in the observation window). As shown in Figure 5, a relatively high quantile (τ = 0.85) was selected because it provides a conservative estimate of the drift threshold. The regression equation is

(10) ξ=−6.6223+14.5922R

According to Figure 5, when *R* = 0.75, *ξ* is approximately 4.3. This implies that when *ξ* > 4.3, more than 75% of the near-bed eggs are likely to escape the circulation cells induced by sand dunes and drift downstream.

### 3.3. Effects of Sediment Deposition on Egg Hatching

**Figure 6:**
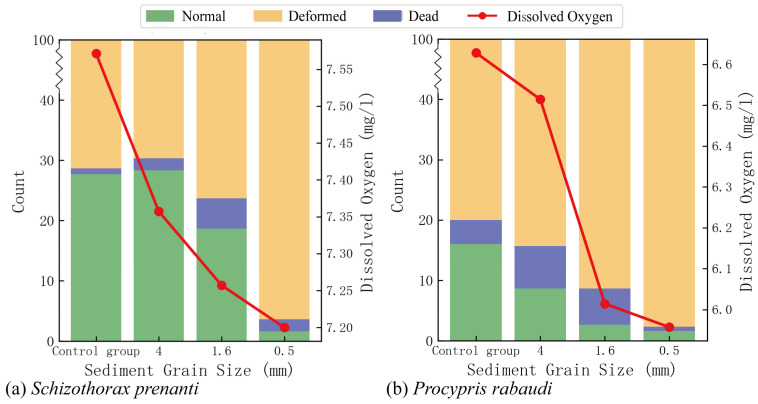
Egg hatching conditions and variations in DO: (**a**) *Schizothorax prenanti*; (**b**) *Procypris rabaudi*.

Figure 6 illustrates the hatching outcomes of *S. prenanti* and *P. rabaudi* when buried by sediment of various grain sizes. Among them, the left vertical axis “count” represents the number of occurrences of different hatching states (normal, deformed, dead, etc.) of *S. prenanti* and *P. rabaudi* under the corresponding sediment grain sizes; the line chart (in red) represents the variation in DO concentration with sediment grain sizes. Overall, sediment deposition significantly reduced the normal hatching rate of both species, particularly under fine-grained sediment conditions. For *S. prenanti* (Figure 6a), the normal hatching rate decreased from 27.6% in the control group to 1.6% at a 0.5 mm grain size. The DO concentration dropped from 7.56 mg/L to 7.20 mg/L. Similarly, for *P. rabaudi* (Figure 6b), the normal hatching rate declined from 16% in the control to 1.6% at 0.5 mm. The DO level decreased more steeply, from 6.60 mg/L to 6.20 mg/L.

To further explore the mechanism by which sediment affects hatching rates, this study employed a mediation analysis by constructing three sets of regression models to examine the effects of sediment on DO, the effects of DO on hatching rates, and the direct and indirect effects of sediment on hatching rates.

Table 4 presents the impacts of sediment deposition on DO, the effects of DO on hatching rates, and the influence of sediment on hatching rates. The results showed that when considering the effects of sediment on hatching rate and DO separately, sediment had a significant negative impact on both (*p* < 0.01, Model 1 and Model 3); fine-grained sediment significantly reduced both the DO levels and the hatching rates of fish eggs (*p* < 0.01, Model 1). When the effects of both sediment and DO on the hatching rate were considered simultaneously, DO exhibited a significant positive effect on the hatching rate, whereas the direct effect of sediment on the hatching rate was not significant (*p* > 0.05, Model 2), indicating that sediment primarily influences hatching outcomes indirectly through its impact on DO.

The decomposition results of the mediation effect further support this explanation (Table 5). For both *P. rabaudi* and *S. prenanti*, the analysis consistently revealed that DO plays a pivotal and decisive role in determining hatching success, whereas the direct effect of sediment was comparatively limited or even negligible.

In summary, influences fish egg hatching rates mainly through its impact on DO levels, highlighting the pivotal role of oxygen availability in embryo development.

## 4. Discussion

### 4.1. Low-Velocity Compensatory Mechanisms and Implications for Flow Management

This study focuses primarily on hydrodynamic thresholds for drifting eggs. The survival of the eggs, however, is also influenced by many other physico-chemical factors.

Firstly, vertical gradients in water quality parameters such as temperature and DO do not always favor eggs in surface layers. In low-flow stream channels with limited vertical mixing, bottom waters may offer more stable temperatures and reduced physical disturbance—either from wind or navigation—conditions that can be more conducive to embryo development [43,44,45,46]. In some cases, local DO concentrations near the bed may even exceed those at the surface [45,46]. As such, eggs that settle near the bed are not necessarily exposed to degraded water quality; under certain conditions, they may in fact benefit from a more stable and favorable physiological environment.

Secondly, the near-bed zone in natural rivers is typically characterized by highly complex turbulence structures shaped by bedform roughness, gravel patches, and microtopographic variability [47]. This dynamic “turbulence workshop” facilitates localized DO exchange and may delay hypoxic stress or even allow settled eggs to be resuspended [33,45,48]. For example, installing structures such as low weirs and spillways in dam or tailwater areas can enhance turbulence near the riverbed, increase DO concentrations, and thereby improve conditions for egg incubation [49]. These findings suggest that, even under hydrodynamically limited scenarios, manipulating non-flow factors can enhance the ecological function of near-bed environments and open new avenues for habitat management.

Moreover, while egg settlement generally leads to reduced hatching success, not all settled eggs are lost. Experiments have shown that in reservoir backwater zones affected by flow impoundment, egg mortality can rise to 45–68%, compared to 15–25% in natural river reaches [50]. Nonetheless, a significant proportion of eggs still hatch successfully. This indicates that, as long as the eggs remain viable, improvements in local hydrodynamic conditions can allow them to re-enter the drift phase [33,51]. At the Three Gorges Reservoir, for example, regular ecological flow releases—short-term water level increases designed to stimulate near-bed disturbance—have proven effective in promoting the hatching of previously settled eggs [52]. These ecological operations have demonstrated tangible benefits for fish recruitment.

In summary, although the near-bed zone is often regarded as a high-risk area for drifting eggs under low-flow conditions, it still holds ecological potential due to compensatory effects of water quality and micro-scale turbulence. From a management perspective, it is important to move beyond the simplistic assumption that “settlement equals failure”, and instead recognize the recoverability and ecological value of this zone. Well-designed ecological flow operations—especially those timed during critical hatching periods—can enhance egg survival and support dual objectives of biodiversity conservation and regulated water supply in managed river systems.

### 4.2. Sediment Deposition Affects Fish Egg Hatching by Altering DO

The substrate is one of the key habitat factors for the spawning of certain fish, and suitable substrate conditions have a significant impact on the egg hatching [53,54,55,56,57]. Previous studies have found that sediment deposition leads to a reduction in the hatching rate of fish eggs [27,28]. This study further clarifies the indirect mechanism by which sediment deposition affects the hatching of adhesive eggs, i.e., DO reduction.

The hatching process is highly sensitive to oxygen. Embryos require a continuous oxygen supply in early development to maintain energy metabolism [58,59]. Although sediment itself may not directly affect the eggs, it indirectly has a significant effect on the hatching rate by altering the DO environment [60,61]. The model results of this study indicate that DO plays a decisive mediating role in the relationship between sediment and the hatching rate.

Numerous studies have shown that fine particles of sediment easily fill the pores in gravel or sand beds, significantly weakening the permeability of the substrate and the material exchange between the water column and the sediment–water interface [62]. When the proportion of fine sand in the bed exceeds a critical threshold (about 30%), water flow through the pores is severely blocked, creating an oxygen-deficient “microhypoxic” environment within the bed layer [62]. In addition, the smaller the particle size, the lower the bed porosity and permeability, making it more difficult for metabolic waste to be removed from the embryos, thereby forming a microenvironment that inhibits embryo development [63,64].

It should be noted that this study reveals the key pathway through which sediment deposition indirectly affects the hatching rate of adhesive fish eggs via DO, emphasizing the central role of DO as a mediator. However, fish eggs have different oxygen demands at various developmental stages, and hypoxic resting eggs can revive when oxygen is restored. Additionally, environmental factors such as water temperature also influence the fish egg’s demand for DO [58,59]. Therefore, future research should focus on the DO requirements during different stages of embryo development and under varying environmental conditions.

## 5. Conclusions

From the perspective of fish reproduction, this study focuses on two key issues: the re-entrainment of drifting fish eggs and the impact of sediment deposition on the hatching of adhesive eggs. Through controlled laboratory experiments, the research systematically explored the re-entrainment patterns of drifting eggs and the mechanisms by which sediment deposition affects the hatching success of adhesive eggs. The results further reveal the pathways through which reservoir groups influence fish reproductive processes, providing scientific support for ecological protection and fishery resource restoration and contributing to the healthy development of river ecosystems. The main findings are as follows:

(1) Model eggs, with a density of 1.008 g/cm^3^ and diameters of 6 mm and 5 mm, were fabricated to simulate the drifting behavior of fish eggs. Through experiments, this study fitted the coefficient in the egg settling velocity formula as *k* = 0.553.
(2) Using dimensional analysis (Π theorem), a threshold formula for near-bed drifting eggs was derived, i.e., $\xi =\alpha \frac{{U_{b}}^{2}DR_{e}}{\frac{r_{s}-r}{r}g\lambda h_{s}}$. This formula comprehensively reflects the motion characteristics of drifting eggs under hydrodynamic forces.
(3) Taking *S. prenanti* and *P. rabaudi* as examples, the study clarified the impact of sediment deposition on the hatching of adhesive eggs and its underlying mechanisms. The results showed that sediment deposition significantly reduced the hatching rate of adhesive eggs, mainly through changes in DO levels.

## Figures and Tables

**Table 1:** Design of two types of model dunes.

Dune	F	L	A	a
Large	0.2	18	1.8	0.782
Small	0.2	9	0.9	0.782

**Table 2:** Experimental scenarios for near-bed drift.

Test Condition	Slope	Egg Diameter (mm)	Dune Size
1	6.07%	5	Large
2	6.07%	5	Small
3	6.07%	6	Large
4	6.07%	6	Small
5	8.20%	5	Large
6	8.20%	5	Small
7	8.20%	6	Large
8	8.20%	6	Small
9	10.79%	5	Large
10	10.79%	5	Small
11	10.79%	6	Large
12	10.79%	6	Small
13	13.03%	5	Large
14	13.03%	5	Small
15	13.03%	6	Large
16	13.03%	6	Small
17	15.45%	5	Large
18	15.45%	5	Small
19	15.45%	6	Large
20	15.45%	6	Small

**Table 3:** Predicted settling velocity of model eggs.

Egg Diameter	k	Free Settling Velocity (m/s)		Relative Difference (%)
		Egg Diameter: 6 mm	Egg Diameter: 5 mm	
Current study	0.553	0.011994	0.010949	
Zhang Xun (2022) [34]	0.735	0.015941	0.014552	+28.9–32.7%
Zhang Kanrui (2022) [41]	0.609	0.013208	0.012058	+8.7–9.1%

**Table 4:** Effects of sediment deposition with different grain sizes on DO and egg hatching rate.

Species	Model	Variable	Coefficient	Standard Error	t	*p*
*P. rabaudi*	Model 1	Intercept	6.1021	0.076	80.167	0.000
		Sediment deposition	0.0227	0.006	3.781	0.004
	Model 2	Intercept	−1.0981	0.378	−2.905	0.017
		DO	0.1923	0.062	3.106	0.013
		Sediment deposition	0.0010	0.002	0.537	0.604
	Model 3	Intercept	0.0751	0.020	3.692	0.004
		Sediment deposition	0.0053	0.002	3.333	0.008
*S. prenanti*	Model 1	Intercept	7.2409	0.016	459.198	0.000
		Sediment deposition	0.0135	0.001	10.899	0.000
	Model 2	Intercept	−15.2564	2.930	−5.207	0.001
		DO	2.1313	0.405	5.267	0.001
		Sediment deposition	−0.0237	0.006	−4.159	0.002
	Model 3	Intercept	0.1759	0.039	4.548	0.001
		Sediment deposition	0.0051	0.003	1.685	0.123

Note: Model 1: effect of sediment on DO; model 2: combined effect of DO and sediment on hatching rate; Model 3: effect of sediment on hatching rate.

**Table 5:** Contribution rates of sediment deposition and DO to fish egg hatching rate.

Species	Effect Type	Estimated Value	Standard Error	t	*p*
*P. rabaudi*	Indirect effect	0.1292	0.0416	3.1052	0.0112
	Direct effect	0.0010	0.0018	0.5370	0.6043
	Total effect	0.1302	0.0416	3.1258	0.0122
	Contribution rate of DO	99.24%	-	-	-
	Direct contribution rate of sediment	0.76%	-	-	-
*S. prenanti*	Indirect effect	0.7907	0.1501	5.2664	0.0004
	Direct effect	−0.0237	0.0057	−4.1588	0.0025
	Total effect	0.7670	0.1502	5.1046	0.0006
	Contribution rate of DO	103.09%	-	-	-
	Direct contribution rate of sediment	−3.09%	-	-	-

## Data Availability

Data are contained within the article.

## References

[1] Chen Q., Li Q., Lin Y., Zhang J., Xia J., Ni J., Cooke S.J., Best J., He S., Feng T. (2023). River Damming Impacts on Fish Habitat and Associated Conservation Measures. Rev. Geophys..

[2] He F., Zarfl C., Tockner K., Olden J.D., Campos Z., Muniz F., Svenning J.-C., Jähnig S.C. (2024). Hydropower impacts on riverine biodiversity. Nat. Rev. Earth Environ..

[3] Csiki S.J., Rhoads B.L. (2014). Influence of four run-of-river dams on channel morphology and sediment characteristics in Illinois, USA. Geomorphology.

[4] Kuriqi A., Pinheiro A.N., Sordo-Ward A., Bejarano M.D., Garrote L. (2021). Ecological impacts of run-of-river hydropower plants—Current status and future prospects on the brink of energy transition. Renew. Sustain. Energy Rev..

[5] Li T., Wang S., Liu Y., Fu B., Zhao W. (2018). Driving forces and their contribution to the recent decrease in sediment flux to ocean of major rivers in China. Sci. Total Environ..

[6] Cao W., Chang J., Qiao Y., Duan Z. (2007). Fish Resources of Early Life History Stages in Yangtze River.

[7] Zhu L., Chen C., Zhang J. (2016). Study on variations of runoff and sediment and effect to the lower Jinsha River. J. Sediment Res..

[8] Zhu L., Chen D., Yang C., Chen K., Li S. (2023). Sediment deposition of cascade reservoirs in the lower Jinsha River and scouring of river channel under dam. J. Lake Sci..

[9] Du Z., Dong X., Zhang F., Qin L. (2022). Study on runoff and sediment characteristics and reservoir deposition in Xiluodu Reservoir of the Jinsha River. J. Sediment Res..

[10] Yue L. (2021). The Evolution of Flow and Sediment Regime in the Yangtze River and Its Fish Response Mechanism. Ph.D. Thesis.

[11] Liu Q., Zhang P., Li H., You L., Li Y., Li J., Liu M., Zhao P., Wang K., Zhu Z. (2021). Assessment and conservation strategies for endemic fish with drifting eggs threatened by the cascade barrier effect: A case study in the Yalong River, China. Ecol. Eng..

[12] Garcia T., Jackson P.R., Murphy E.A., Valocchi A.J., Garcia M.H. (2013). Development of a Fluvial Egg Drift Simulator to evaluate the transport and dispersion of Asian carp eggs in rivers. Ecol. Model..

[13] Yu Z.T., Liang Z.S., Yi B.L. (1984). The early development of *Coreius heterodon* and *Coreius guichenoti*. Acta Hydrobiol. Sin..

[14] Li T., Tang L., Wang L., An L., Wang J., Mo K.L., Chen Q.W. (2020). Distribution characteristics and ecological types changes in fish communities under hydropower development from Xiluodu to Xiangjiaba reach. Acta Ecol. Sin..

[15] Zhang P., Liu Q., Wang Y., Li K., Qin L., Liang R., Li J. (2022). Does drifting passage need to be linked to fish habitat assessment? Assessing environmental flow for multiple fish species with different spawning patterns with a framework integrating habitat connectivity. J. Hydrol..

[16] Stanley J.G., Miley W.W., Sutton D.L. (1978). Reproductive requirements and likelihood for naturalization of escaped grass carp in the United States. Trans. Am. Fish. Soc..

[17] Leslie A.J., Van Dyke J.M., Nall L.E., Miley W.W. (1982). Current velocity for transport of grass carp eggs. Trans. Am. Fish. Soc..

[18] Kolar C.S., Chapman D., Courtenay W.R., Housel C.M., Williams J.D., Jennings D.P. (2007). Bigheaded Carps: A Biological Synopsis and Environmental Risk Assessment.

[19] Kocovsky P.M., Chapman D.C., McKenna J.E. (2012). Thermal and hydrologic suitability of Lake Erie and its major tributaries for spawning of Asian carps. J. Great Lakes Res..

[20] Li C. (2012). A Preliminary Analysis of the Impacts of the Cascade Hydropower Development on the Fish Biodiversity in the Upper Reach of the Yangtze River. Master’s Thesis.

[21] George A.E., Chapman D.C., Deters J.E., Erwin S.O., Hayer C.A. (2015). Effects of sediment burial on grass carp, *Ctenopharyngodon idella* (Valenciennes, 1844), eggs. J. Appl. Ichthyol..

[22] Lu Y., Zhu W.-Y., Liu Q.-Y., Li Y., Tian H.-W., Cheng B.-X., Zhang Z.-Y., Wu Z.-H., Qing J., Sun G. (2022). Impact of Low-Head Dam Removal on River Morphology and Habitat Suitability in Mountainous Rivers. Int. J. Environ. Res. Public Health.

[23] Wei Q. (2003). Reproductive Behavioral Ecology of Chinese Sturgeon (*Acipenser sinensis* Gray) with Its Stock Assessment. Ph.D. Thesis.

[24] Qiang J., Zhong C.Y., Bao J.W., Liang M., Liang C., Tao Y.F., Li H.X., He J., Xu P. (2019). Synergistic effect of water temperature and dissolved oxygen concentration on rates of fertilization, hatching and deformity of hybrid yellow catfish (*Tachysurus fulvidraco*♀ × *Pseudobagrus vachellii*♂). J. Therm. Biol..

[25] Guo J., Liao B., Zhou Q., Nie R. (2020). Experimental study on influence of sediment concentration to survival of *Schizothorax prenanti*. J. Sediment Res..

[26] Qin X.H., Liu G.Y., Wu Y.J., Shi X.T., Wang X.L. (2017). Effects of Light Intensity on the Hatching Rate of Fertilized *Schizothorax chongi* Eggs and on the Growth and Feeding of Larvae. J. Hydroecol..

[27] Chojnacki K.A., George A.E., DeLonay A.J. (2023). The effects of substrate and sediment burial on survival of developing pallid sturgeon (*Scaphirhynchus albus*) and shovelnose sturgeon (*S. platorynchus*) embryos. Environ. Biol. Fishes.

[28] Julien H. (2000). The Impact of Winter Fluvial Processes on Spawning Grounds and Intergranular Survival of Atlantic Salmon (*Salmo salar* L.). Master’s Thesis.

[29] Kähler C.J., Astarita T., Vlachos P.P., Sakakibara J., Hain R., Discetti S., La Foy R., Cierpka C. (2016). Main results of the 4th International PIV Challenge. Exp. Fluids.

[30] Guo J. Modelling river bedform evolution. Proceedings of the 15th International Symposium on River Sedimentation.

[31] Wang Q., Han Y., Li P., Zhang W., Wang Y., Xi Y., Yao W. (2023). Ecohydraulic modelling to evaluate cascade dam construction impact and support fish habitat restoration. Ecol. Eng..

[32] Zhang X., Zhang K., Yang W., Wang L., Yang S., Li W., Zhang P. (2022). Hydrostatic Settling Characteristics of Drifting Fish Eggs in Main Stream of Yangtze River from Yibin to Fengdu. Resour. Environ. Yangtze Basin.

[33] Garcia T., Zamalloa C.Z., Jackson P.R., Murphy E.A., Garcia M.H. (2015). A Laboratory Investigation of the Suspension, Transport, and Settling of Silver Carp Eggs Using Synthetic Surrogates. PLoS ONE.

[34] Zhang X. (2022). Study on the Movement Characteristics of Drifting Fish Eggs in the Upper Reaches of the Yangtze River. Ph.D. Thesis.

[35] Wang B.H., Wang X.Z. (1996). New Method for Calculation of Settling Velocity of a Spherodial Particle. China Powder Sci. Technol..

[36] Xiong M.H., Shao K., Li W.T., Zhu B. (2023). Research progress on resources variation and protection of *Coreius guichenoti*. Yangtze River.

[37] Liu L., Wu G., Wang Z. (1990). Reproduction ecology of *Coreius heterodon* (Bleeker) and *Coreius guichenoti* (Sauvage et Dabry) in the mainstream of the Changjiang River after the construction of Gezhouba Dam. Acta Hydrobiol. Sin..

[38] Ren Y., Zhao L., Cao H., Ruan Y. (2020). Influence of ecological regulation of cascade reservoirs in the lower Jinsha River. Ecol. Environ. Monit. Three Gorges.

[39] Zhang D., Fan H., Wang M., Li F., Ruan Y. (2022). Target Fish Screening for the Ecological Operation of Wudongde Hydropower Station on Jinsha River. J. Hydroecol..

[40] Yang Z., Zhang P., Tang H., Gong Y., Dong C., Chen X., Zhao N. (2017). The formation of habitat suitability curves for *Coreius guichenoti* (Sauvage & Dabry de Thiersant, 1874) of the lower Jinsha River. Ecol. Sci..

[41] Zhang K. (2023). Study on Drifting Fish Egg Movement Patterns in Non-Uniform Water Flows. Ph.D. Thesis.

[42] Curtis W., Logan J.D., Parker W. (1982). Dimensional analysis and the pi theorem. Linear Algebra Appl..

[43] Evans E., Petts G.E. (1997). Hyporheic temperature patterns within riffles. Hydrol. Sci. J..

[44] Sawyer A.H., Bayani Cardenas M., Buttles J. (2012). Hyporheic temperature dynamics and heat exchange near channel-spanning logs. Water Resour. Res..

[45] Higashino M., O’Connor B.L., Hondzo M., Stefan H.G. (2008). Oxygen transfer from flowing water to microbes in an organic sediment bed. Hydrobiologia.

[46] Reeder W.J., Quick A.M., Farrell T.B., Benner S.G., Feris K.P., Tonina D. (2018). Spatial and temporal dynamics of dissolved oxygen concentrations and bioactivity in the hyporheic zone. Water Resour. Res..

[47] Shumilova O.O., Sukhodolov A.N., Constantinescu G.S., MacVicar B.J. (2021). Dynamics of shallow wakes on gravel-bed floodplains: Dataset from field experiments. Earth Syst. Sci. Data.

[48] Grant S.B., Gomez-Velez J.D., Ghisalberti M. (2018). Modeling the effects of turbulence on hyporheic exchange and local-to-global nutrient processing in streams. Water Resour. Res..

[49] Dressing S.A. (2003). National Management Measures to Control Nonpoint Source Pollution from Agriculture.

[50] Hu P., Tang J., Yang Z., Zeng Q., Yang M. (2021). Experimental study on critical hydrodynamic conditions for safe drifting of semi-buoyant eggs. J. Hydraul. Eng..

[51] Liu X., Lin J., Peng Q., Yu K., Chen Y., Zhuang J. (2018). Experimental research on fish eggs’ movement using particle tracking velocimetry technique. J. Hydraul. Eng..

[52] Jun X., Pan L., Qian C. (2024). Water supply safety and technological innovation safeguarding for the Three Gorges Project. China Water Resour..

[53] Du H., Wei Q., Zhang H., Liu Z., Wang C., Li Y. (2011). Bottom substrate attributes relative to bedform morphology of spawning site of Chinese sturgeon *Acipenser sinensis* below the Gezhouba dam. J. Appl. Ichthyol..

[54] Duerregger A., Pander J., Palt M., Mueller M., Nagel C., Geist J. (2018). The importance of stream interstitial conditions for the early-life-stage development of the European nase (*Chondrostoma nasus* L.). Ecol. Freshw. Fish.

[55] Merz J.E., Setka J.D., Pasternack G.B., Wheaton J.M. (2004). Predicting benefits of spawning-habitat rehabilitation to salmonid (*Oncorhynchus* spp.) fry production in a regulated California river. Can. J. Fish. Aquat. Sci..

[56] Chapman J.M., Proulx C.L., Veilleux M.A., Levert C., Bliss S., Andre M.-E., Lapointe N.W., Cooke S.J. (2014). Clear as mud: A meta-analysis on the effects of sedimentation on freshwater fish and the effectiveness of sediment-control measures. Water Res..

[57] Hao D., Qiwei W., Hui Z., Chengyou W., Jinming W., Li S. (2015). Changes of bottom substrate characteristics in spawning ground of Chinese sturgeon downstream the Gezhouba dam from impounding of Three Gorge reservoir. Acta Ecol. Sin..

[58] Kochhann D., Chapman L. (2023). A foundational exploration of respiration in fish eggs and larvae. Fish Physiology.

[59] Harter T. (2021). Warm fish eggs gasp for oxygen. J. Exp. Biol..

[60] Decent Q. (2020). Factors Controlling Dissolved Oxygen in Spawning Gravels: Evaluation of the Sediment Intrusion and Dissolved Oxygen Model (SIDO) for Fisheries Management. Ph.D. Thesis.

[61] Broman E., Brüsin M., Dopson M., Hylander S. (2015). Oxygenation of anoxic sediments triggers hatching of zooplankton eggs. Proc. R. Soc. B Biol. Sci..

[62] Chen K., Guo Z., Zhan Y., Roden E.E., Zheng C. (2024). Heterogeneity in permeability and particulate organic carbon content controls the redox condition of riverbed sediments at different timescales. Geophys. Res. Lett..

[63] Shrivastava S., Stewardson M., Arora M. (2021). Sediment reworking in streambeds with fine sediment deposits and its influence on hyporheic flow regime. Water Resour. Res..

[64] Greig S., Sear D., Carling P. (2005). The impact of fine sediment accumulation on the survival of incubating salmon progeny: Implications for sediment management. Sci. Total Environ..

